# Host Stress Response Is Important for the Pathogenesis of the Deadly Amphibian Disease, Chytridiomycosis, in *Litoria caerulea*

**DOI:** 10.1371/journal.pone.0062146

**Published:** 2013-04-22

**Authors:** John D. Peterson, John E. Steffen, Laura K. Reinert, Paul A. Cobine, Arthur Appel, Louise Rollins-Smith, Mary T. Mendonça

**Affiliations:** 1 Department of Biological Sciences, Auburn University, Auburn, Alabama, United States of America; 2 School of Biological Sciences, Washington State University, Pullman, Washington, United States of America; 3 School of Science, Penn State Erie, Erie, Pennsylvania, United States of America; 4 Department of Pathology, Microbiology and Immunology, Vanderbilt University Medical Center, Nashville, Tennessee, United States of America; 5 Department of Entomology and Plant Pathology, Auburn University, Auburn, Alabama, United States of America; 6 Departments of Pathology, Microbiology and Immunology and of Pediatrics, Vanderbilt University Medical Center and Department of Biological Sciences, Vanderbilt University, Nashville, Tennessee, United States of America; Imperial College Faculty of Medicine, United Kingdom

## Abstract

Chytridiomycosis, a disease caused by *Batrachochytrium dendrobatidis*, has contributed to worldwide amphibian population declines; however, the pathogenesis of this disease is still somewhat unclear. Previous studies suggest that infection disrupts cutaneous sodium transport, which leads to hyponatremia and cardiac failure. However, infection is also correlated with unexplained effects on appetite, skin shedding, and white blood cell profiles. Glucocorticoid hormones may be the biochemical connection between these disparate effects, because they regulate ion homeostasis and can also influence appetite, skin shedding, and white blood cells. During a laboratory outbreak of *B. dendrobatidis* in Australian Green Tree Frogs, *Litoria caerulea*, we compared frogs showing clinical signs of chytridiomycosis to infected frogs showing no signs of disease and determined that diseased frogs had elevated baseline corticosterone, decreased plasma sodium and potassium, and altered WBC profiles. Diseased frogs also showed evidence of poorer body condition and elevated metabolic rates compared with frogs showing no signs of disease. Prior to displaying signs of disease, we also observed changes in appetite, body mass, and the presence of shed skin associated with infected but not yet diseased frogs. Collectively, these results suggest that elevated baseline corticosterone is associated with chytridiomycosis and correlates with some of the deleterious effects observed during disease development.

## Introduction

Emerging infectious diseases (EIDs) of wildlife can have profound effects on animal biodiversity [1], [2]; however, little is known about the pathogenesis of most wildlife EIDs [3]. Since wildlife EIDs are often associated with anthropogenic and environmental stressors, pathogenesis is likely influenced by the host’s response to stressors [3]–[6]. The evolutionarily conserved stress response is one of the mechanisms by which vertebrates modulate responses to these stressors [7]. The stress response is of interest in a disease context, because it is mediated by glucocorticoid (GC) hormones that are known to affect susceptibility to infection [8].

GCs influence a suite of physiological functions in vertebrates, including reproduction, development, blood ion homeostasis, metabolism, appetite, growth, and, importantly in the context of disease, immunity [9]. While much is known about how GCs influence physiological function in non-diseased animals, much less is known about how GCs influence the same physiological functions in diseased animals. To our knowledge only one such study has been conducted in wild vertebrates. Warne et al. [10] exposed *Rana sylvatica* to ranaviruses and observed an increase in corticosterone (CORT; the most abundant amphibian GC) concentration and accelerated developmental changes consistent with the effects of endogenous and exogenous elevations of CORT in non-diseased amphibians.

Chytridiomycosis, a disease caused by the amphibian chytrid fungus *Batrachochytrium dendrobatidis* (*Bd*) [11] has contributed to worldwide amphibian population declines. It is considered to be a significant threat to global amphibian biodiversity [2], [12]–[14]. Chytridiomycosis, like CORT, influences blood ion homeostasis, appetite, skin shedding, and immunity. Specifically, *Bd* disrupts sodium transport in the host’s epidermis, which leads to hyponatremia and cardiac failure [15]. *Bd* also suppresses appetite [15], [16], disrupts normal skin shedding [15], [16], and causes alterations in white blood cell (WBC) abundance [17], [18]. Yet there are no studies that have attempted to document what hormones may be mediating these changes in blood ions, behavior, shedding, and WBC abundance.

GCs may mediate the aforementioned effects of *Bd* infection. In amphibians, GCs are critical regulators of blood ion homeostasis [19]–[24], appetite [25], [26], skin shedding [27]–[30], and WBC numbers and immune function [31]–[37]. A normal, adaptive, regulatory mechanism to maintain sodium homeostasis is likely a moderate, transitory elevation in CORT secretion to increase cutaneous uptake of sodium as well as digestive uptake (facilitated by increased appetite) [23], [24], [26]. Because *Bd* infection directly compromises cutaneous sodium transport, a sustained elevation of CORT could occur to maintain ion homeostasis. However, high concentrations of GCs may become maladaptive, altering immune responses [36]–[39], increasing metabolic rate [40], as well as actually suppressing appetite [41]. The suppression of appetite may further exacerbate ion imbalance, leading to unsustainable blood sodium levels and cardiac failure. Thus, CORT, in its regulatory role of maintaining ion homeostasis, may be elevated in response to disease caused by infection, and could contribute to *Bd*-induced mortality.

The overall aims of our study were to determine whether *Bd* infection influences CORT levels and whether CORT profiles are associated with previously undescribed, as well as previously described effects of *Bd* infection. We documented the relationship of *Bd* infection to plasma CORT, sodium, and potassium concentrations; food intake; skin shedding; and WBC profiles during an outbreak of *Bd* in a laboratory colony of Australian Green Tree Frogs (*Litoria caerulea*). Because both CORT and disease influence energy balance, we also monitored resting metabolic rate (RMR), body condition, and body mass.

## Results

### Evaluation of Pre-diseased Frogs

**Figure 1:**
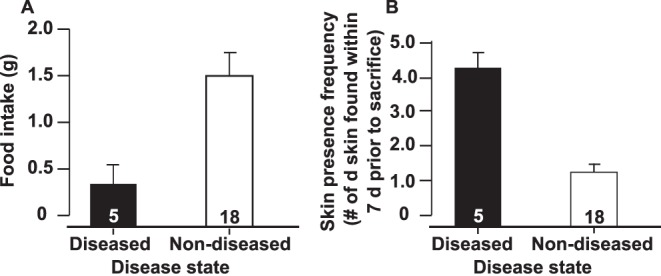
Evaluation of pre-diseased frogs. Average food intake (Fig. 1A) and skin presence frequency (Fig. 1B) +1 standard error of *Litoria caerulea* that eventually became diseased or remained non-diseased at one week prior to sacrifice (Fig. 1A) and within the week leading up to sacrifice (Fig. 1B). Disease states were statistically different (Food intake: ANOVA, P = 0.022, F_1,21_ = 6.16; skin presence frequency: ANOVA, P<0.001, F_1,21_ = 38.11).

**Figure 2:**
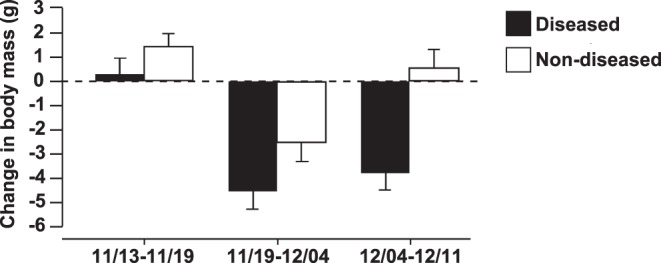
Change in body mass throughout infection. Average change in body mass (±1 standard error) of *Litoria caerulea* that eventually became diseased (n = 9) or remained non-diseased (n = 14) for chytridiomycosis between dates leading up to sacrifice on 12/12/09. Disease states were statistically different (Repeated measures ANOVA, Disease status: P<0.001, F_1,21_ = 38.06, Time: P<0.001, F_1,42_ = 12.25, Disease status × Time: P = 0.2).

Individuals that eventually displayed clinical signs of disease (e.g. listlessness, odd body posture, and skin discoloration) consumed significantly less food one week prior to displaying clinical signs of disease compared to individuals that displayed no signs of disease (ANOVA, P = 0.022, F_1,21_ = 6.16; Fig. 1). During the week prior to sacrifice, shed skin was found on significantly more days within bins of frogs that eventually became diseased than within the bins of non-diseased frogs (ANOVA, P<0.001, F_1,21_ = 38.11; Fig. 1). Frogs that eventually became diseased also lost significantly more weight than frogs that remained non-diseased in the weeks prior to sacrifice (Repeated measures ANOVA, Disease status: P<0.001, F_1,21_ = 38.06, Time: P<0.001, F_1,42_ = 12.25, Disease status × Time: P = 0.2; Fig. 2).

### Evaluation of Diseased Frogs

**Figure 3:**
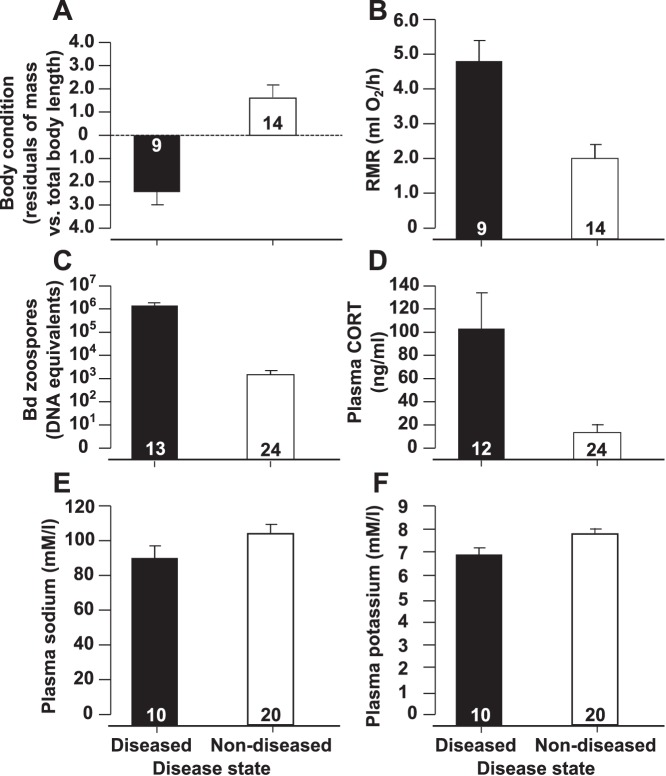
Evaluation of diseased frogs. Mean body condition (Fig. 3A), resting metabolic rate (RMR, Fig. 3B), log transformed *Bd* zoospore equivalents +1 (Fig. 3C), plasma corticosterone (CORT, Fig. 3D), plasma sodium (Fig. 3E), and plasma potassium (Fig. 3F) ±1 standard error for *Litoria caerulea* that displayed clinical signs of disease (diseased) or did not display clinical signs of disease (non-diseased). Disease states were significantly different for all measures (ANOVA/ANCOVA, P<0.05). Sample sizes vary among measures because of sampling limitations.

Approximately 24 hours prior to sacrifice, frogs that displayed clinical signs of chytridiomycosis had significantly lower body conditions (ANCOVA, Disease status: P<0.001, F_1,20_ = 19.02; Total body length: P<0.001, F_1,20_ = 273.43). There was no disease status by total body length interaction (P = 0.22). For ease of interpretation, these data are visually presented as average residuals from a regression of body mass by total body length (Fig. 3). Diseased individuals also consumed significantly more oxygen compared to non-diseased frogs (ANCOVA, Disease status: P<0.001, F_1,20_ = 24.52, Body mass: P = 0.010, F_1,20_ = 7.99; Fig. 3). There was no disease status by body mass interaction (P = 0.3).

When sacrificed, swabs taken from diseased frogs contained significantly more *Bd* zoospore equivalents than swabs taken from non-diseased individuals (ANOVA, P<0.001, F_1,35_ = 19.66; Fig. 3). Although non-diseased individuals all had detectable levels of *Bd*, they contained approximately 1,000 times fewer zoospore equivalents than diseased individuals, on average.

**Figure 4:**
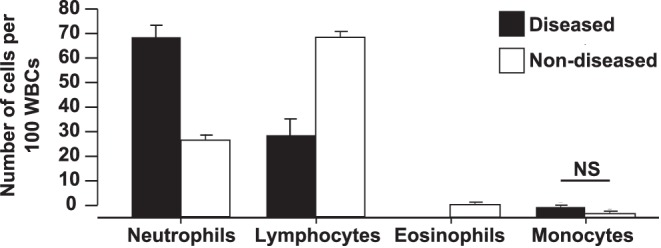
White blood cell counts of diseased frogs. Average relative abundance of neutrophils, lymphocytes, eosinophils, and monocytes per 100 white blood cells (WBC) +1 standard error from *Litoria caerulea* displaying clinical signs of chytridiomycosis (diseased, n = 7) or not displaying clinical signs of disease (non-diseased, n = 19). WBC abundance profiles were significantly different between disease states (MANOVA, P<0.001, F_1,20_ = 12.26). Blood smears from diseased frogs contained significantly fewer lymphocytes and eosinophils and significantly more neutrophils than smears from non-diseased frogs (Sheffe’s range test, P<0.001). Disease states had similar numbers of monocytes (NS) and basophils (not shown).

Blood parameters determined at sacrifice also differed with disease status. Diseased frogs contained significantly fewer plasma electrolytes (ANOVA, Sodium: P = 0.049, F_1,28_ = 4.24, Potassium: P = 0.049, F_1,28_ = 4.24; Fig. 3) and significantly greater concentrations of plasma CORT (ANOVA, P = 0.001, F_1,34_ = 18.73; Fig. 3) compared with non-diseased frogs. Additionally, WBC profiles differed significantly between diseased and non-diseased individuals (MANOVA, P<0.001, F_1,20_ = 12.26; Fig. 4). Blood smears from diseased frogs contained significantly fewer lymphocytes and eosinophils and significantly more neutrophils among 100 WBCs counted than smears from non-diseased frogs (Sheffe’s range tests, P≤0.002).

### Changes in CORT, RMR, and Lymphocyte Abundance at different Bd Burdens

**Figure 5:**
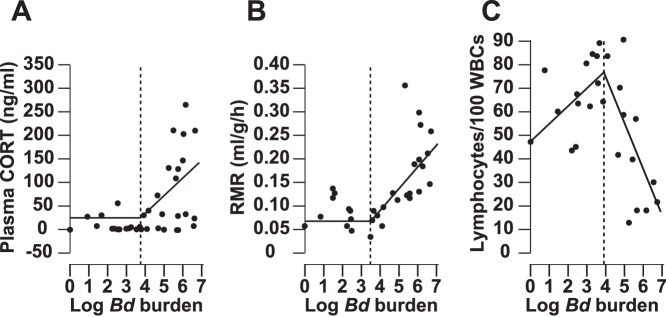
Changes in variables at different ***Bd*** **burdens.** Segmented regressions of plasma corticosterone (CORT, Fig. 5A), resting metabolic rate (RMR, Fig. 5B), and lymphocyte abundances (Fig. 5C) of *Litoria caerulea* during an outbreak of chytridiomycosis. Horizontal and vertical dotted lines indicate X and Y axes, respectively. Vertical dashed lines indicate breakpoints at which the dependent variables changed significantly. Black lines indicate the two segments fit to the data before and after the breakpoint. The zoospore breakpoints for CORT, RMR, and lymphocytes were 4,940; 4,066; and 10,778 zoospores, respectively. Data before and after the breakpoint were significantly different for all three variables (Segmented regression, P<0.05). Linear regressions suggested significant relationships between *Bd* burden and CORT (R^2^ = 0.25, P = 0.002, F_1,35_ = 11.40), RMR (R^2^ = 0.46, P<0.001, F_1,22_ = 18.16), and lymphocytes (R^2^ = 0.20, P = 0.022, F_1,25_ = 5.96).

Because all individuals in the study contained different *Bd* burdens and were, thus, apparently at different points in infection we used segmented regression to estimate the zoospore intensity at which CORT, RMR, and lymphocyte abundances changed significantly (the zoospore breakpoints). The zoospore breakpoints for CORT, RMR, and lymphocytes were 4,940; 4,066; and 10,778 zoospores, respectively (Fig. 5). Linear regressions suggested significant relationships between *Bd* burden and CORT (R^2^ = 0.25, P = 0.002, F_1,35_ = 11.40), RMR (R^2^ = 0.46, P<0.001, F_1,22_ = 18.16), and lymphocytes (R^2^ = 0.20, P = 0.022, F_1,25_ = 5.96).

## Discussion

Individuals that displayed clinical signs of chytridiomycosis had significantly elevated baseline CORT, decreased plasma sodium and potassium, altered WBC profiles, increased RMR, and decreased body condition compared with non-diseased individuals. WBC profiles and RMR changes in diseased frogs parallel those observed following treatment with glucocorticoids in other studies (e.g. increased neutrophils and oxygen consumption and decreased lymphocytes and eosinophils in relation to total WBC) [31]–[35], [40], [42]. It is important to note that non-diseased individuals were also infected, but released thousands of *Bd* zoospores while diseased individuals released millions of zoospores, on average. When we plotted this broad range of *Bd* burdens (regardless of disease status) against CORT, RMR, and lymphocytes, segmented regressions indicated these three variables changed significantly at similar breakpoints (4,000–10,000 zoospores; Fig. 5).

Appetite suppression likely contributed to other effects we observed. For example, appetite suppression likely exacerbated hyponatremia because amphibians take up sodium via digestive as well as cutaneous routes [43]. Additionally, amphibians usually consume their shed skin, so appetite suppression also could explain why we observed shed skin more often in the containers of frogs that eventually became diseased [43]. Finally, appetite suppression, coupled with an increased RMR, may have contributed to the weight loss and poor body condition observed in frogs that eventually became diseased. With no input of food, sick frogs must catabolize body tissues to meet their elevated respiratory demand, which results in weight loss and reduced body condition.

The levels of CORT in plasma were determined after development of several effects, so although infection was associated with decreased food intake, increased presence of shed skin, and weight loss, it is unclear whether increased CORT secretion was a cause or consequence of these parameters. We happened to be monitoring food intake, skin shedding, and weight loss as part of a separate experiment when the outbreak occurred, thus we did not monitor CORT along with these other variables. Although we observed changes in CORT, RMR, and lymphocytes at similar *Bd* burdens, future studies are needed to determine the timing of when key physiological variables change during infection and whether CORT manipulation can alter pathogenesis.

It is unclear whether infection-induced GC secretion is beneficial or maladaptive in vertebrates. Few studies have tested the effects of disease on GC levels in vertebrates [44]–[57]. Even fewer have observed how this may then lead to beneficial or deleterious physiological effects. To our knowledge, this is the first study that has assessed the effects of disease on baseline CORT levels in an adult amphibian (however CORT levels have been assessed in tadpoles) [10], [56], [57]. Our data suggest that disease, at least chytridiomycosis, may be a potent modulator of baseline plasma CORT. Frogs displaying clinical signs of disease contained eight times more plasma CORT than non-diseased frogs. Average plasma CORT was 104 ng/ml in symptomatic frogs (maximum level of 270 ng/ml), rivaling the highest average levels of CORT observed in amphibians in response to other stressors [58]–[62]. Given these findings and the large number of emerging diseases in wildlife, we suggest that more studies focus on post-infection stress responses in wild animals.

Better understanding of the physiological effects of CORT, and its involvement in mediating factors that threaten the conservation status of amphibians (e.g. habitat destruction, global climate change, pollution, etc.) is needed. Specifically, there is a lack of data on how CORT influences metabolic rate and appetite in amphibians [25], [63]. Our study provides data to suggest that CORT is associated with these factors, but controlled laboratory data are needed to complement this study. Understanding how amphibians respond to environmental change has become more urgent given recent amphibian population declines [64]. Several perturbations that potentially contribute to amphibian population declines have been linked to stress physiology (e.g. anthropogenic contaminants [62], [65]–[72], disease [10], [56], [57], low habitat quality [73], and habitat desiccation [74]). Though the influence of anthropogenic contaminants on the stress axis has been relatively well studied in amphibians, far less is known about how disease, habitat destruction, invasive predators, and climate change may influence stress physiology. Given the powerful and far reaching effects of GCs on wildlife life histories, understanding how these hormones mediate the interplay between environmental perturbations and life histories is essential to future conservation efforts.

## Materials and Methods

### Ethics Statement

All methods were approved by Auburn University Institutional Animal Care and Use Committee (Permit Number: 2009-1620). All surgery was performed following euthanasia and all efforts were made to minimize suffering.

### Laboratory Outbreak and Experimental Design

Ninety eight *Litoria caerulea* were obtained commercially from an amphibian trader (Tri Reptile, Miami, FL) who imported the frogs from Indonesia, in autumn of 2009. Within a month, the frogs became ill and died at a rapid rate (i.e., 13 died within 13 days). Shed skin from individuals showing clinical signs of chytridiomycosis (e.g., listlessness, odd body posture, and skin discoloration) [75] was viewed under a light microscope and *Bd* was detected by visual inspection of the skin in all samples viewed. It is unclear whether infection originated from the wild or from our laboratory.

At this point, disease status (i.e., individuals displaying clinical signs of chytridiomycosis [diseased] or individuals not displaying clinical signs [non-diseased]) was monitored daily and food intake was assessed in all remaining non-diseased individuals (n = 79). When an individual became diseased, the diseased frog and two randomly selected non-diseased individuals were swabbed (to quantify *Bd* zoospores), pithed, and bled (within 3 min of handling). Several drops of whole blood were used to make blood smears for enumeration of WBCs. The remaining blood was centrifuged for 4 min at 3,500×g and the plasma was frozen and stored at −20°C for later use in ion and CORT assays.

### Animal Care

Amphibians were housed individually in plastic containers (17×17×17 cm) in which paper towels saturated with well water were used as substrate. Wet paper towels were replaced twice each week for the duration of the study. Light was provided by full spectrum light bulbs on a 12∶12 light/dark cycle. Room temperature was maintained by a thermostat at ∼22°C.

### Bd Zoospore Burden

Frogs were swabbed in a standardized fashion by lightly brushing a sterile cotton swab (Medical Wire & Equipment, MW113) 10 times over the sides, venter, and ventral surface of the thighs and 5 times over the underside of each foot [76]. Zoospore equivalents were determined by standard extraction and quantitative PCR techniques [77], [78]. Nucleic acids were extracted by adding 60 µl of PrepMan Ultra (Applied Biosystems, Foster City, CA) and 30–35 mg of Zirconium/silica beads (0.5 mm, Biospec Products, Bartlesville, OK) to the tip of each swab. Samples were homogenized for 45 s in a Mini Beadbeater (MP Bio, Solon, OH) and centrifuged for 30 s at 15,000×g. After a second homogenization and centrifugation, the samples were boiled for 10 min, returned to room temperature for 2 min, and centrifuged at 15,000×g for 3 min. Nucleic acids in the supernatant were removed for real-time PCR. Samples were loaded into an Mx3000P Real-Time PCR system (Stratagene, La Jolla, CA) for 40 cycles of 95°C for 10 min, 95°C for 30 s, 55°C for 1 min, and 72°C for 1 min. Zoospore equivalents were determined using a standard curve and indicate *Bd* burden.

### Ion Analyses

Plasma prepared by centrifugation of whole blood for 4 min at 3,500×g to remove red blood cells, was analyzed by Inductively Coupled Plasma with Optical Emission Spectroscopy (ICP-OES, Perkin Elmer 7100 DV, Waltham, MA) with simultaneous measurement of Ca, Co, Cu, Fe, K, Mg, Mn, Mo, Na, P, S, Zn [79]. Equal volume of plasma was diluted into ultra-pure, metal-free water (MilliQ, Millipore) then centrifuged at 13,000×g to remove particulates and then introduced directly into the instrument argon plasma using a cyclonic nebulizer. Metal concentrations are determined comparing emission intensities to a standard curve created from certified metal standards (SPEX, Metuchen, NJ). Standard curves were confirmed by re-analysis of standard solutions diluted in a matrix equivalent to the sample. Individual readings are the average of two intensity measurements varied by less than 5%. Repeated analysis of individual samples showed less than 5% variability.

### Radioimmunoassay

Plasma CORT concentrations were determined by radioimmunoassay as described by Mendonça et al. [80]. All samples were run in one assay. Extraction efficiency was 81% and intraassay variation was 19.8%.

### Food Intake and Body Mass

Frogs were weighed weekly from the time they arrived in the laboratory. Once a week, for two weeks prior to sacrifice, each animal was blotted dry, weighed, and fed approximately 10% of their body weight with 2.5 week old crickets coated in vitamin dust. All crickets not consumed were weighed after 24 hours. Food intake was determined as the mass of the crickets not consumed subtracted from the original mass of crickets given to the frog.

### Shed Skin Collection

A subsample of frogs and bins were examined daily for the presence of shed skin. Shed skin was removed if it was observed on amphibians or within their containers. Dates in which skin was found on a frog or within its container were recorded. When frogs were sacrificed, the number of days, within the previous seven days, the frogs had shed skin on their body or within their container was determined. This value is referred to hereafter as “skin presence frequency”.

### Relative WBC Numbers

Dried blood smears were stained with a Hema 3 kit (Fisher scientific, Kalamazoo, MI) and viewed under a light microscope. Slides were read in a standard zig-zag fashion. One hundred WBCs were observed and the number of neutrophils, lymphocytes, eosinophils, monocytes, and basophils were recorded.

### Respirometry and Body Condition

Closed system respirometry was used to measure RMR (oxygen consumption) 1 day prior to sacrifice following the methods of Ward et al. [81]. Frogs were first acclimated in their respirometry chamber (140 ml syringes served as the respirometry chambers, Monoject, Sherwood Medical Industries, Ballymoney, UK) for at least 45 min in a darkened incubator (22°C). For the respirometry measurements, the frogs were then incubated for ∼50 min in a darkened incubator (22°C). Any frogs that urinated or defecated during incubation were excluded from analyses. Frogs were weighed and their total body length was determined following respirometry to determine body condition [82].

### Statistical Analyses

*Bd* burden; plasma CORT, sodium, and potassium; food intake; and skin presence frequency were compared relative to disease status with analyses of variance (ANOVAs). RMR was compared between disease states (i.e. diseased or non-diseased) with an analysis of covariance (ANCOVA) with disease status as the independent variable, RMR as the dependent variable, and body mass as the covariate. Since body mass influences RMR, RMR is presented as least squared means, corrected for body mass. Body condition was estimated as the residuals obtained by regressing body mass against total body length. Body condition was compared between disease states with an ANCOVA, with disease status as the independent variable, body mass as the dependent variable, and total body length as the covariate. Change in body mass was compared relative to disease status with a repeated measures ANOVA, because the same individuals were sampled multiple times. Relative WBC numbers were compared relative to disease status with a multivariate analysis of variance (MANOVA). Sheffe’s range tests were conducted for all a posteriori comparisons. We were unable to monitor changes over time for several variables; however, when diseased and non-diseased frogs were sacrificed *Bd* burden was highly variable across all frogs (ranging from 0 to millions of zoospores per frog), suggesting that each frog was at a different point within disease progression. We used segmented regression to determine the threshold *Bd* burden at which the trend of CORT, RMR, and lymphocytes changed significantly (breakpoints), regardless of disease status [83]. We also used linear regression to determine whether there were linear relationships between *Bd* burden and CORT, RMR, and lymphocytes. SAS (SAS institute, version 9.2) was used for the RMR ANCOVA (PROC GLM) and all segmented regressions (PROC NLIN). StatView for Windows (SAS institute, version 5.0.1) was used for all other statistical analyses. All data met the assumptions of normality and significance was determined as P≤0.05.
